# Carbolic Acid in Carbuncle

**Published:** 1885-12

**Authors:** N. B. Kennedy

**Affiliations:** Hillsboro, Texas


### CARBOLIC ACID IN CARBUNCLE.

HILLSBORO, TEXAS, Nov. 21st, 1885.

EDITOR DANIEL'S TEXAS MEDICAL JOURNAL,
Austin, Texas.

*Dear Sir :* I see in the last number of your valuable Journal, that C. H. Wilkinson, M. D., of Galveston, says, in reference to "Carbolic Acid Injections in the Treatment of Carbuncle :" "In its presentation, therefore, I can confidently claim originality in its conception, and prolonged study in its evolution," etc.

I beg leave to state that I first used carbolic acid *hypodermi-*

*cally* for the cure of carbuncles, hæmorrhoids, tumors, snake bites, and foul and ill-conditioned ulcers, in 1875. And have used it successfully ever since.

I made known my treatment in a paper read before the Texas State Medical Association, which convened in the city of Waco, Texas, April, 1881, entitled "The Hypodermic Administration of Carbolic Acid, for the Cure and Removal of Foul and Ill-Conditioned Ulcers, both Internally and Externally Situated; Poisonous Bites, Hæmorrhoids, Carbuncles, and Tumors." Said paper met with much opposition, and was openly condemned by some of the leading lights in the profession, at the time it was read. I was unavoidably absent, on account of professional engagements, but fortunately for me the paper was ably championed by that accomplished gentleman and profound scholar, A. M. Douglass, M. D., of Covington, Texas.

While I would not detract from Dr. Wilkinson, yet at the same time I wish most emphatically to be placed on record as being the originator and first person to use the remedy for the above mentioned diseases. Wishing long life to your splendid Journal, I am,

Yours truly,

N. B. Kennedy, M. D.

